# Methane Emission Reduction and Biological Characteristics of Landfill Cover Soil Amended With Hydrophobic Biochar

**DOI:** 10.3389/fbioe.2022.905466

**Published:** 2022-06-08

**Authors:** Yongli Qin, Beidou Xi, Xiaojie Sun, Hongxia Zhang, Chennan Xue, Beibei Wu

**Affiliations:** ^1^ Guangxi Key Laboratory of Environmental Pollution Control Theory and Technology, Guilin University of Technology, Guilin, China; ^2^ School of Life and Environmental Sciences, Guilin University of Electronic Technology, Guilin, China; ^3^ Guangxi Collaborative Innovation Center for Water Pollution Control and Water Safety in Karst Area, Guilin University of Technology, Guilin, China; ^4^ State Environmental Protection Key Laboratory of Simulation and Control of Groundwater Pollution, Chinese Research Academy of Environmental Sciences, Beijing, China

**Keywords:** landfill cover, biochar, hydrophobic, methane emission reduction, biological characteristics

## Abstract

Biochar-amended landfill cover soil (BLCS) can promote CH_4_ and O_2_ diffusion, but it increases rainwater entry in the rainy season, which is not conducive to CH_4_ emission reduction. Hydrophobic biochar–amended landfill cover soil (HLCS) was prepared to investigate the changes in CH_4_ emission reduction and biological characteristics, and BLCS was prepared as control. Results showed that rainwater retention time in HLCS was reduced by half. HLCS had a higher CH_4_ reduction potential, achieving 100% CH_4_ removal at 25% CH_4_ content of landfill gas, and its main contributors to CH_4_ reduction were found to be at depths of 10–30 cm (upper layer) and 50–60 cm (lower layer). The relative abundances of methane-oxidizing bacteria (MOB) in the upper and lower layers of HLCS were 55.93% and 46.93%, respectively, higher than those of BLCS (50.80% and 31.40%, respectively). Hydrophobic biochar amended to the landfill cover soil can realize waterproofing, ventilation, MOB growth promotion, and efficient CH_4_ reduction.

## 1 Introduction

Methane (CH_4_) is an important greenhouse gas, and its global warming potential is 28-fold that of CO_2_ (Malyan et al., 2016). A large amount of CH_4_ is released during the degradation of domestic wastes in the landfill (Feng et al., 2019; Ghosh et al., 2019). Technologies that reduce CH_4_ emissions mainly include resource utilization and *in situ* emission reduction (Lou et al., 2011; Di Trapani et al., 2019). Methane oxidation of landfill cover as an *in situ* emission reduction technology is mostly used in small-sized, medium-sized, and aging landfills (Sadasivam and Reddy, 2014). Methane-oxidizing microorganisms play a vital role in the landfill cover (Mancinelli and McKay, 1984).

Clay is a widely used covering material because of its low price, wide source, non-toxicity, and easy construction (Christophersen et al., 2001; Chanton et al., 2009), but it is prone to crack formation, has limited diffusion of CH_4_ and O_2_, and lacks nutrients, thus restricting its application in landfill cover (Chanton et al., 2011a, 2011b; Scheutz et al., 2011a). Researchers currently use biological cover materials (Scheutz et al., 2011b; Lou et al., 2011), one of which is biochar that attracts attention due to its high porosity, large specific surface area, and high biological affinity (Sadasivam, 2015; Xu et al., 2016; Bamdad et al., 2019; Cimon et al., 2020). Yaghoubi (2011) studied the CH_4_ adsorption capacity of soil, biochar, and soil containing 10% and 20% biochar and found maximum values of 32, 346, 59, and 82 ml kg^−1^, respectively, indicating that the CH_4_ adsorption capacity of landfill cover soil increases with the biochar content. Reddy et al. (2014) suggested that the number of methane-oxidizing bacteria (MOB) in biochar-amended soil column is higher than that in soil alone. Huang et al. (2019) found that a 15% volume ratio for biochar amendment in landfill cover soil enhances CH_4_ removal efficiency, thus revealing the possible advantages of biochar amendment in terms of CH_4_ emission reduction, such as high porosity conducive to the diffusion of CH_4_ and O_2_ and great retention of nutrients for MOB.

In the biochar-amended cover soil, the hydraulic conductivity increases with the biochar content, 2% biochar addition increases the hydraulic conductivity by an order of magnitude (Reddy et al., 2021). An increase in hydraulic conductivity will cause a large amount of rainwater to enter the biochar-amended cover soil during rainy season (Wong et al., 2022), thereby occupying pores of the cover soil, increasing the moisture content of the cover soil and negatively affecting CH_4_ adsorption and oxidation (Scheutz et al., 2009; Yargicoglu and Reddy, 2017). Increasing the biochar content in the cover soil is necessary to promote the entry of CH_4_ from the landfill and O_2_ from the atmosphere into the cover soil, thereby increasing the efficiency of methane oxidation for the biochar-amended cover soil. However, rainwater can be prevented from entering by reducing the proportion of biochar, which leads to a decrease in the CH_4_ adsorption and oxidation efficiency of the system. Therefore, promoting the diffusion of CH_4_ and O_2_ and preventing rainwater from entering the cover layer have become the key to the application of biochar landfill cover soil technology (Cheng et al., 2018).

The development of hydrophobic biochar that can achieve waterproof and breathable properties will provide a way to solve the aforementioned problems (Sun et al., 2019; Wu et al., 2020). In this study, hydrophobic biochar replaced ordinary biochar and was added to the landfill cover soil to form hydrophobic biochar–amended landfill cover soil (HLCS). A biochar-amended landfill cover soil (BLCS) control group was also prepared. The CH_4_ emission reduction performance and biological characteristics of HLCS were investigated to provide a theoretical basis for the HLCS application.

## 2 Materials and Methods

### 2.1 Preparation of Hydrophobic Biochar–Amended Landfill Cover Soil

Hydrophobic biochar was made by our laboratory through modifying rice straw biochar with hydrophobic modifier silane-coupling agent KH570 (CH_2_=C(CH_3_)COOC_3_H_6_Si(OCH_3_)); the related preparation process have been published (Sun et al., 2019). The water absorption of hydrophobic biochar is 1.27 g (g biochar)^−1^, only 20% of that of rice straw biochar.

HLCS was prepared based on the results of our previous study for BLCS (Qin et al., 2021), by mixing the hydrophobic biochar with the landfill cover soil at a volume ratio of 1:4. A control group BLCS was also provided. Physical and chemical properties of BLCS and HLCS as specified in Table 1. There were no significant differences in maximum compaction dry density and nutritional indicators (such as N content, K content, and organic matter content) of BLCS and HLCS. The plasticity index of HLCS was obviously lower than that of BLCS, indicating that HLCS contained fewer hydrophilic groups (Soltani-Jigheh et al., 2019).

**TABLE 1:** Physical and chemical properties of biochar-amended and hydrophobic biochar–amended landfill cover soils.

Properties	Biochar-amended soil	Hydrophobic biochar–amended soil
Maximum compaction dry density (g cm^−3^)	1.56	1.58
Plasticity index (%)	21	9
pH	8.18	7.46
P (%)	0.1	0.09
K (%)	0.19	0.21
Organic matter content (%)	14.75	14.93

### 2.2 Experimental Device

**FIGURE 1:**
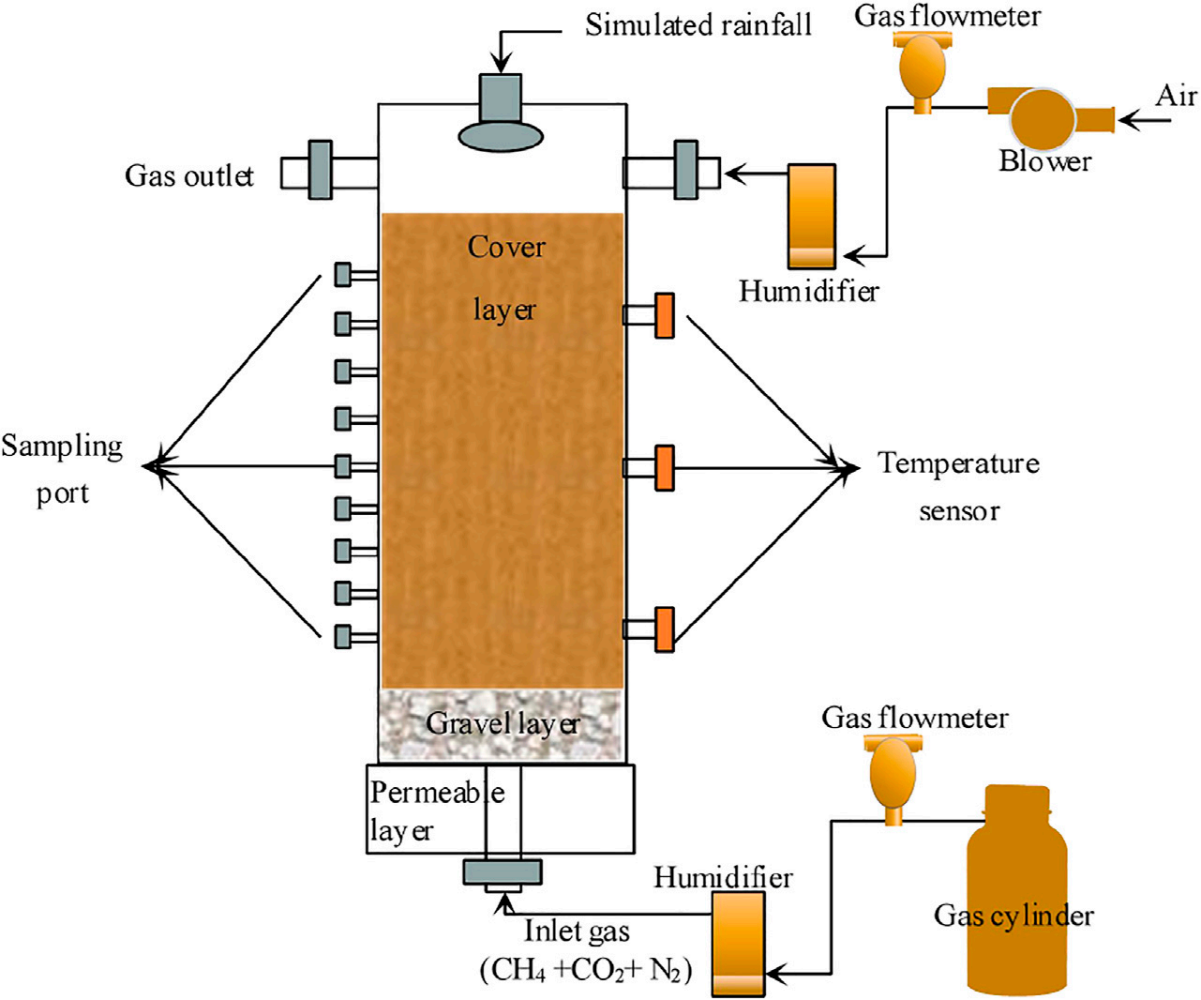
Schematic of the landfill cover–simulated column.

Two landfill cover–simulated columns (CB and CH) made of plexiglass with 100-cm height and 15-cm diameter were set up for the CH_4_ emission reduction experiment (Figure 1). Air was blown in from the top of the column by a blower (flow rate = 50 ml min^−1^), and simulated landfill gas composed of CH_4_, CO_2_, and N_2_ in a certain proportion was pumped into the bottom of the column (flow rate = 15 ml min^−1^) (Reddy et al., 2014). Air and landfill gas were humidified into the column to add moisture. The simulated columns were composed of cover, gravel, and permeable layers from top to bottom. The height of cover layer was 60 cm (Yargicoglu and Reddy, 2018). For the two simulated columns CB and CH, the cover materials consisted of biochar-amended and hydrophobic biochar–amended landfill cover soils, respectively. The relative compaction of two columns was approximately 80% (Reddy et al., 2014). The initial moisture content of two columns was 20%. In total, nine sampling ports were set on one side of the cover layer to collect gas and soil samples numbered 1–9 from top to bottom, and the interval between each sampling port was 5 cm. The height of the gravel layer was 10 cm to support the cover layer. The permeable layer with a height of 15 cm can discharge the excess moisture of the cover layer.

### 2.3 Operation Method

The simulated columns of CB and CH were operated by increasing the content of CH_4_ in the simulated landfill gas. The whole operation process consisted of the following three stages: stage Ⅰ (days 1–30) with CH_4_ content of 5%, stage Ⅱ (days 31–60) with CH_4_ content of 15%, and stage Ⅲ (days 61–95) with CH_4_ content of 25%. The experiment was finished after the CH_4_ was almost completely removed.

### 2.4 Sampling and Analysis Methods

The gas inlet and outlet of simulation columns were closed during sampling. Gas samples at each stage were periodically collected by syringes from the outlets, 1# (upper cover layer, 10 cm depth), 5# (middle cover layer, 30 cm depth), and 9# (lower cover layer, 50 cm depth) of CB and CH into collection bags for CH_4_ content analysis. Each gas sample included 20 ml of gas in a collection bag. The degree of contribution of CH_4_ removal at different depths in the simulation column depends on the amount of CH_4_ removal at that depth, that is, the differential between the CH_4_ content at the inlet and outlet of that depth. The total CH_4_ removal rate was determined by measuring the differential between the CH_4_ content of the inlet and outlet of the simulation column at regular time intervals.

$${\text{The total CH}}_{4}\;\text{removal rate }=\;\frac{C_{in}-C_{out}}{C_{in}},$$

where *C*_*in*_ and *C*_*out*_ are the CH_4_ content of the inlet and outlet of the simulation column (%), respectively.

Soil samples were collected by sterile spoons from the 1#, 5#, and 9# sampling ports of simulation columns at the beginning of the operation (day 1) and the end of stages Ⅰ, Ⅱ, and Ⅲ (days 30, 60, and 95, respectively) into sterile tubes to study the changes in microbial community structure. Each soil sample included 2 g of soil in a sterile tube. The soil samples collected from the 1#, 5#, and 9# sampling ports at the beginning (day 1) of CB were named B1.1, B1.5, and B1.9, and those collected at the end of the stage I were named B2.1, B2.5, and B2.9. The naming rules of the soil samples collected from CH were the same as those of CB. The collected soil samples were qualified by DNA extraction, PCR amplification, product purification, library preparation, and library inspection and then sequenced on NovaSeq6000 (Beijing Nuohe Zhiyuan Technology Co., Ltd.). The primers used for PCR amplification of bacteria were 341F_806R and Arch519F_915R for archaea. R and Origin software were used to visualize the data sets of the microbial community characteristics after quality control, OTU clustering, and species annotation.

At the end of stage Ⅲ, 2 g of soil samples from each of the upper, middle, and lower cover layers of CB and CH were taken with sterilized spoons for fluorescence *in situ* hybridization (FISH). The probes used in the FISH experiment are shown in Table 2.

**TABLE 2:** 16S r RNA-targeted oligonucleotide probes used in fluorescence *in situ* hybridization.

Probe	Sequence	Target microorganism	Fluorescent dyes	Color	References
Eub338Ⅰ	5′-GCT​GCC​TCC​CGT​AGG​AGT-3′	Eubacteria	AMCA	Blue	Zhang et al., 2010
Eub338Ⅱ	5′-GCA​GCC​ACC​CGT​AGG​TGT-3′		AMCA	Blue	
Eub338Ⅲ	5′-GCT​GCC​ACC​CGT​AGG​TGT-3′		AMCA	Blue	
Mγ84	5′-CCA​CTC​GTC​AGC​GCC​CGA-3′	Type Ⅰ MOB	FAM	Green	Eller et al., 2001
Mγ705	5′-CTG​GTG​TTC​CTT​CAG​ATC-3′		FAM	Green	
Mγ450	5′-ATC​CAG​GTA​CCG​TCA​TTA​TC-3′	Type Ⅱ MOB	CY5	Red	

## 3 Results and Discussion

### 3.1 Methane Emission Reduction of the Landfill Cover

**FIGURE 2:**
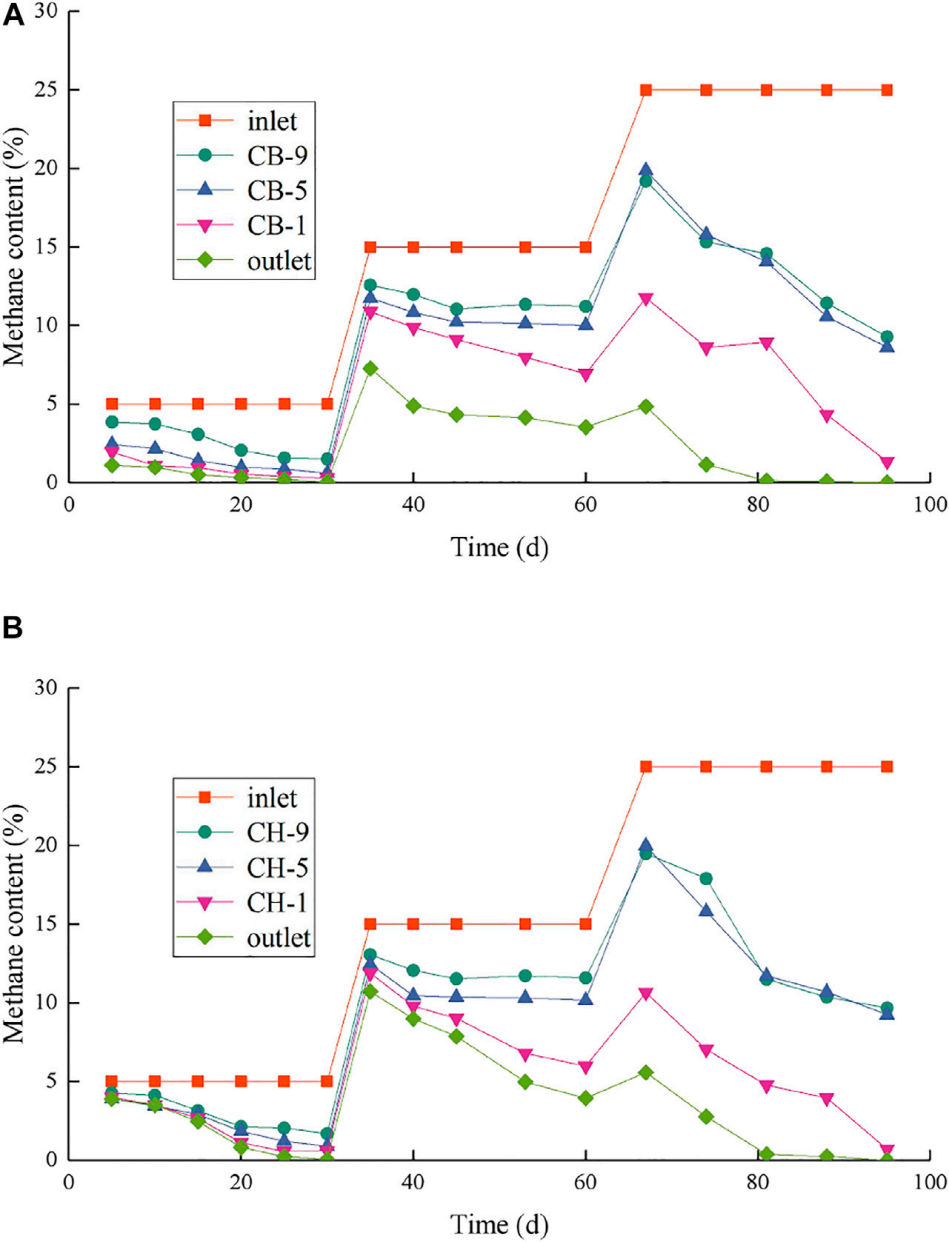
Changes of CH_4_ content in sampling ports of CB **(A)** and CH **(B)** at different stages.

CB and CH were operated by continuously increasing the CH_4_ content of simulated landfill gas. The whole process was divided into three stages and lasted for 95 days. Changes in CH_4_ content at each sampling port of CB and CH during the operation are shown in Figure 2.

In stage Ⅰ (days 1–30), the CH_4_ content in the simulated gas for CB and CH was 5%. The CH_4_ content of each sampling port gradually decreased with the operation of simulated column. On day 5, the CH_4_ contents of outlet of CB and CH were 1.12% and 3.90%, respectively, and the total removal rate of CH_4_ of CB and CH were 77.60% and 22.00%, respectively. The CH_4_ removal rate of CH was lower at the initial stage compared with that of CB possibly because the microorganisms have not fully adapted to the hydrophobic biochar environment. The CH_4_ removal efficiency of CB and CH was remarkably improved with further operation of the simulated column. On day 30, the total removal rates of both CB and CH reached over 98%, but CH was still slightly lower than CB. The CH_4_ content in the simulated landfill gas was then further increased to 15%, and the experiment entered stage Ⅱ (days 31–60). The CH_4_ removal efficiency at the beginning of stage Ⅱ was lower compared with that in stage Ⅰ possibly because the microorganisms have not adapted to the high CH_4_ content. The CH_4_ removal efficiency of each simulated column was remarkably improved with further domestication. At the end of the stage Ⅱ (day 60), the CH_4_ contents of 9#, 5#, 1#, and outlet of CB were 11.21%, 10.01%, 6.93%, and 3.54%, respectively, and the CH_4_ contents of 9#, 5#, 1#, and outlet of CH were 11.58%, 10.16%, 5.98%, and 3.94%, respectively. The main contributors of CB and CH to CH_4_ removal were extended from depth 50–60 cm (below nine# sampling port) in stage Ⅰ to the depths 50–60 cm and 10–30 cm (between 1# and 5# sampling ports) in stage Ⅱ, while the depth 0–30 cm of the traditional landfill cover soil is the main part responsible for CH_4_ removal due to the limited of oxygen (Feng et al., 2019). It can be seen that the CH_4_ removal of CB and CH was not limited by oxygen with the addition of high-porosity biochar (Huang et al., 2019). The total CH_4_ removal rate of CH (73.73%) was still slightly lower than that of CB (76.40%). But the CH_4_ content of 1# sampling port of CH was significantly lower than that of CB in the later stage Ⅱ (days 45–60), and the CH_4_ removal efficiency at the depth 10–30 cm of CH was higher than that of CB (Figure 2), indicating that the CH_4_ oxidation performance of CH was further improved.

In stage Ⅲ (days 61–95), the CH_4_ content in the simulated landfill gas of CB and CH were elevated to 25%. The CH_4_ removal efficiency of CB and CH was not remarkably affected by the increase in CH_4_ content and even showed a growing trend, indicating that the simulation column has gradually stabilized and is not easily affected by load mutation. The CH_4_ removal efficiency at different depths of CB and CH were further improved and then stabilized with further domestication. At the end of the experiment (day 95), the CH_4_ contents of 9#, 5#, 1#, and outlet of CB were 9.28%, 8.61%, 1.35%, and 0.02%, respectively, and the total removal rate of CB was 99.90%, which was higher than that of traditional soil covers (average is 35%) (Christophersen et al., 2001) and woody biochar soil covers (46%–56%) (Reddy et al., 2014; Yargicoglu and Reddy, 2017; Huang et al., 2019), indicating that biochar can provide convenient conditions for the oxidation and adsorption of CH_4_, and different biochar materials may affect the efficiency of CH_4_ reduction (Liu et al., 2015; Wong et al., 2017; He et al., 2018). The CH_4_ contents of 9#, 5#, 1#, and outlet of CH at the 95th day were 9.67%, 9.25%, 0.70%, and 0%, respectively. The total removal rate of CH was 100%, and the CH_4_ removal efficiency at the depth 10–30 cm of CH was significantly higher than that of CB, representing the potential to reduce CH_4_ with higher concentration for CH. The CH_4_ removal of CB and CH were mainly performed at depths of 50–60 and 10–30 cm, respectively, indicating these depths have relatively high methane oxidation activity (Yargicoglu and Reddy. 2018; Reddy et al., 2019) and abundance of MOB (as described in 3.3). As seen before, hydrophobic biochar instead of ordinary biochar in the landfill cover soil can also achieve efficient CH_4_ removal.

### 3.2 Moisture Content and Methane Emission Reduction of Landfill Cover After Simulated Rainfall

A simulated rainfall experiment was conducted to study the waterproof and breathability performance of hydrophobic biochar at the end of stage Ⅲ. Approximately, 2.5 L of water was added to CB and CH through the sprinkler head at the top of columns. The residence time of rainwater in the cover layer was the time between the spraying of sprinkler head and the continuous outflow of water in the permeable layer. The residence time of rainwater was 101.23 s in CB and was shortened by half to 50.55 s in CH, indicating that rainwater stayed in the CH for a shorter time and HLCS has good hydrophobic property.

**FIGURE 3:**
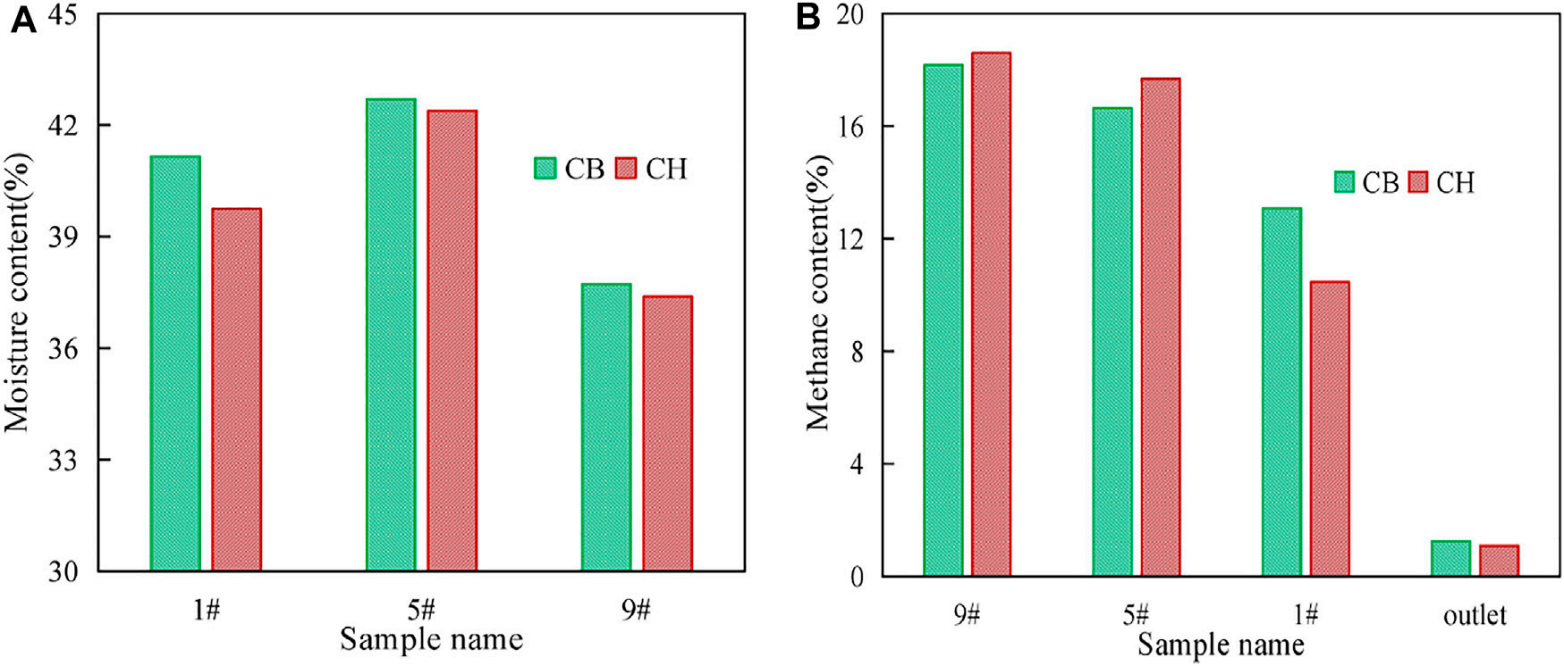
Changes of moisture content **(A)** and methane content **(B)** in CB and CH after simulated rainfall.

The 4 g soil samples and 20 ml gas samples were collected from each of sampling ports 1#, 5#, and 9# of CB and CH after water addition to measure moisture content and CH_4_ removal efficiency, respectively. The changes of moisture and CH_4_ content of CB and CH are shown in Figure 3. After the water addition, the soil moisture contents of 1#, 5#, and 9# of CH were all lower than CB, the CH_4_ removal efficiency of CH was less affected than that of CB, especially for the soil moisture contents of upper layer of CH(1#, 39.75%) was significantly lower than that of CB(1#, 41.15%), and the CH_4_ content of 1# of CH(10.46%) was also significantly lower than that of CB(13.07%). It is worth noting that although HLCS improves the waterproof and breathable properties of the cover layer to reduce CH_4_ emission on rainy days, due to the hydrophobic properties of its material, rainwater is not retained in the cover layer after rainfall, but flows into the landfill layer quickly, resulting in a large amount of leachate generation instantly. Therefore, it is necessary to consider a certain slope and install drainage pipes at the bottom of the slope during the construction of hydrophobic biochar soil landfill cover. In addition, the assessment of the biosafety of KH570 in leachate is also important.

### 3.3 Biological Characteristics of Landfill Cover

#### 3.3.1 Composition of Bacterial Community in the Landfill Cover

**FIGURE 4:**
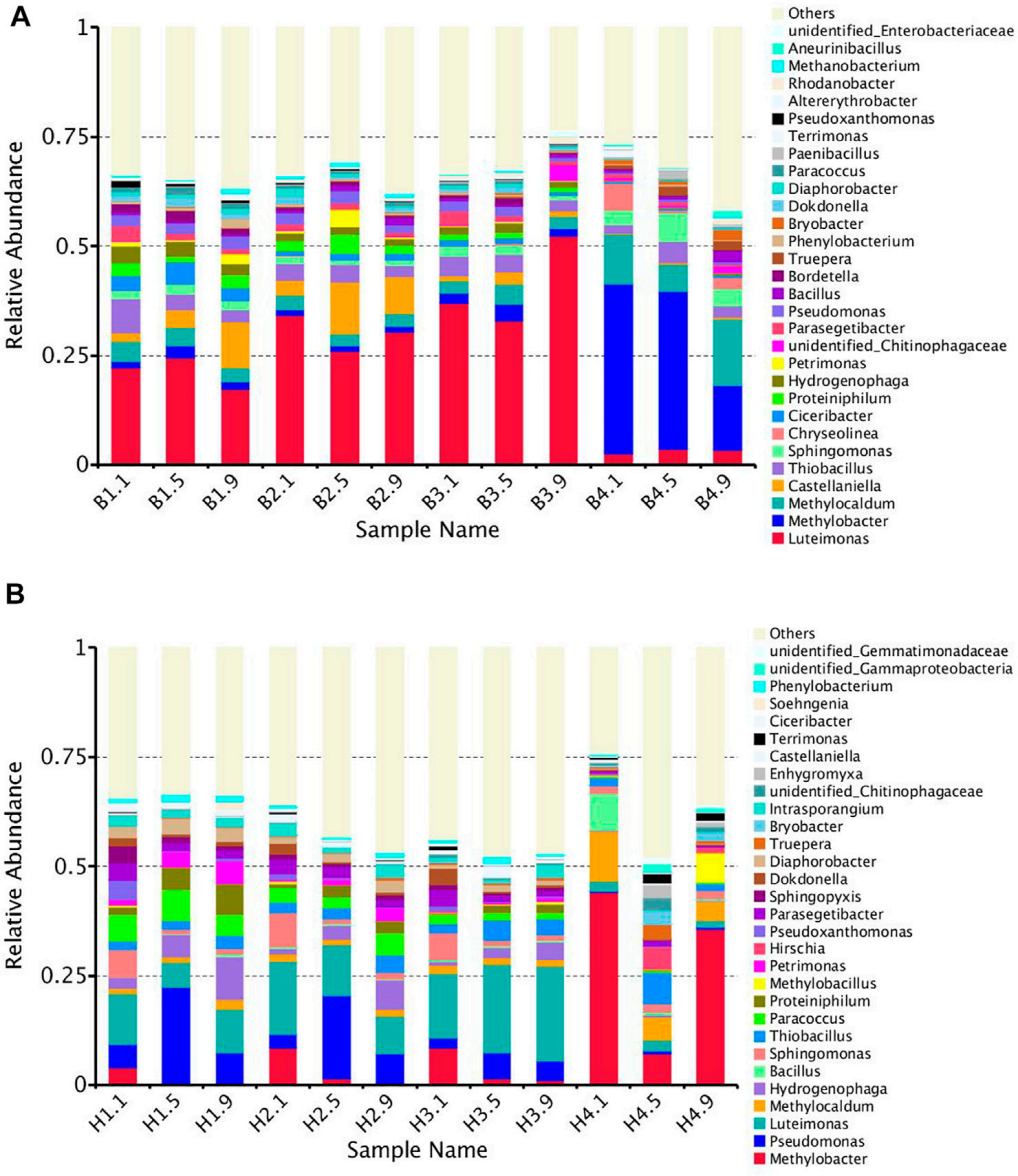
Histogram of species relative abundance at the genus level of CB **(A)** and CH **(B)**.

In total, 24 samples at different depths and stages of CB and CH were analyzed by high-throughput sequencing. The bacterial community composition at genus level (top 30) is shown in Figure 4. At the end of stage Ⅰ and stage Ⅱ, *Luteimonas* had the highest abundance in CB, and then decreased to the lowest in the end of stage Ⅲ (Figure 4A). *Luteimonas* has a strong ability of organic matter degradation (Takashi et al., 2004). The high relative abundance of *Luteimonas* in the early stage may be due to the presence of rich organic matter in the cover that was generated from the decay of some microorganisms that were not suitable for environmental change. The continuous decrease in the relative abundance of some bacteria (*Castellaniella*, *Ciceribacter*, *Proteiniphilum*, *Petrimonas*, etc) in the early stage also confirmed this speculation. The change trend of *Luteimonas* in CH was the same as that of CB, but the relative abundance of *Luteimonas* was significantly lower than CB (Figure 4B). The hydrophobic modifier KH570 was speculated to be an environmentally friendly material (Yang et al., 2012), whereas biochar modified by KH570 was added to soil cover, provided nutrients and suitable moisture for the growth of microorganisms, protected the flora from environmental threats, and reduced the decline of microorganisms (only 10 species of CH declined, while 15 species of CB in the top 30 genera). Some Common bacteria such as *Sphingomonas*, *Bacillus*, *Truepera*, *Bryobacter* and *Terrimonas* were enriched in CB and CH, but the bacteria enriched in different depths of the landfill cover were different, and some different genera were observed with the enrichment in different columns, indicating that different covering materials and different depths would significantly affect the distribution of the bacteria in the landfill cover (Cole et al., 2019; Huang et al., 2019; Wong et al., 2019).

**FIGURE 5:**
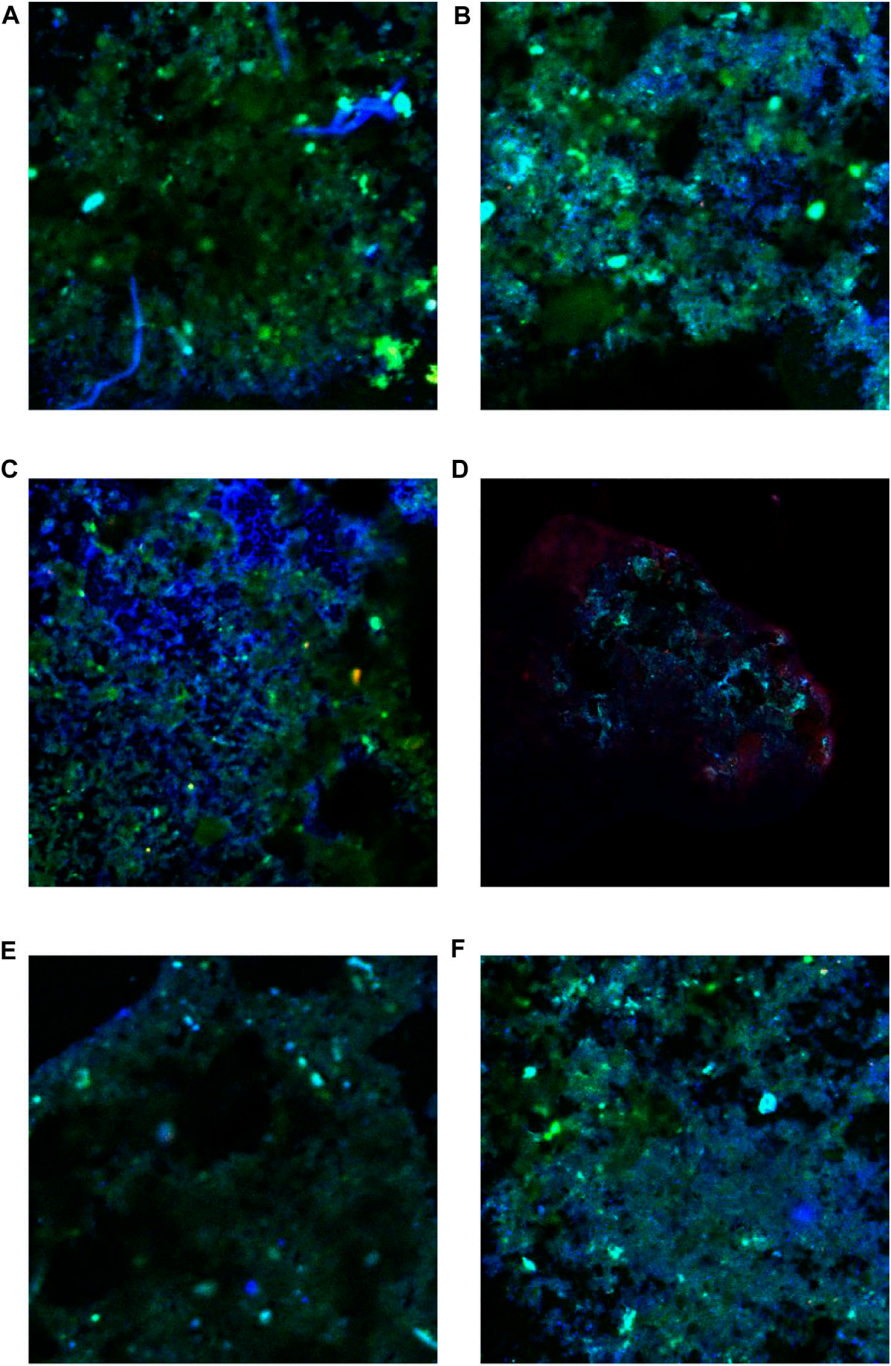
Fluorescence *in situ* hybridization diagram of methane-oxidizing bacteria at each layer of CB and CH. **(A)** Upper layer of CB. **(B)** Upper layer of CH. **(C)** Middle layer of CB. **(D)** Middle layer of CH. **(E)** Lower layer of CB. **(F)** Lower layer of CH.

The MOB was enriched with the increase of CH_4_ content of simulated landfill gas in CB and CH. The distribution of MOB in each landfill cover at the end of the experiment is shown in Table 3. The FISH detection results of CB and CH are shown in Figure 5. Six types of main MOB in CB and CH, including Type Ⅰ *Methylobacter*, *Methylocaldum*, and *Methylloccus*, Type Ⅱ *Methylocystis*, and other types of *Methylobacillus* and *Methyloversatilis,* were detected (Table 3). *Methylocaldum* and *Methylloccus* were also classified among them as Type X (Kalyuzhnaya et al., 2015). The dominant MOB in the upper layer of CB and CH were Type Ⅰ *Methylobacter*, which is the major abundant methanotrophs reported in other studies of landfill cover soil (Chi et al., 2015; Reddy et al., 2019). The green fluorescence representing Type I MOB in the upper layer of CH was more and brighter than that of CB (Figures 5A,B), and the total abundance of MOB in the upper layer of CH(55.93%) was higher than that of CB(50.80%), which was consistent with changes of the CH_4_ removal efficiency in this depth (Figure 2). The dominant MOB in the lower layer of CB were *Methylobacter* and *Methylocaldum*, and for the CH was *Methylobacter*. The relative abundance of total MOB in the lower layer of CH (46.93%) was higher than that for the same depth of CB(31.40%), and with more and brighter green fluorescence in CH (Figures 5E,F). The abundance of total MOB in the lower layers of both CH and CB were lower than that in the upper layers, but the CH_4_ removal rates were significantly higher than those in the upper layers, indicating that the high CH_4_ content in the lower layers were also critical for the improvement of CH_4_ removal efficiency. The dominant MOB in the middle layers of CB and CH were *Methylobacter*. The green fluorescence in the middle layer of CH was weaker and less than that of CB (Figures 5C,D). The relative abundance of total MOB in the middle layer of CH(13.02%) was visibly less than that for the same depth of CB(42.67%), and with little difference in the inlet CH_4_ content (9#) in this layer, it is presumed to be a lower level of immediate CH_4_ production in the middle layer of CH, supported by the lower abundance of methanogenic archaea in the middle and lower layers of CH(as described in Section 3.3.2).

**TABLE 3:** Relative abundance of methane-oxidizing bacteria of different cover layers at the end of the experiment.

Taxonomy	*Methylocaldum* (%)	*Methylobacter* (%)	*Methylococcus* (%)	*Methylobacillus* (%)	*Methylocystis* (%)	*Methyloversatilis* (%)	Sum (%)
B4.1	11.33	38.95	0.19	0.32	0.01	0.00	50.80
B4.5	6.24	35.97	0.25	0.18	0.03	0.00	42.67
B4.9	15.28	14.65	0.59	0.85	0.03	0.00	31.40
H4.1	11.47	44.05	0.20	0.20	0.01	0.00	55.93
H4.5	5.17	7.23	0.27	0.27	0.03	0.05	13.02
H4.9	4.49	35.59	0.13	6.69	0.02	0.01	46.93

From this it is clear that hydrophobic biochar was conducive to MOB growth in upper and lower layers and thereby achieved high-efficiency CH_4_ removal, and the dominant MOB in different cover materials was varied. Some researchers suggested that the high CH_4_ oxidation rates and high MOB abundances were present in the upper layers of amended soil (0–30 cm depth) where the O_2_ availability was high (Reddy et al., 2014; Yargicoglu and Reddy, 2017; Feng et al., 2019). While in this study, the high CH_4_ oxidation rates and high MOB abundances were also present in the lower layer of HLCS. It can be seen that some MOB (such as *Methylobacter*) can also survive in low oxygen conditions (Grinsven et al., 2020), and higher MOB enrichment at the lower layer can be achieved by gradually increasing CH_4_ content of the inlet gas and with long-term acculturation (Reddy et al., 2014).

#### 3.3.2 Composition of Archaea Community in the Landfill Cover

**FIGURE 6:**
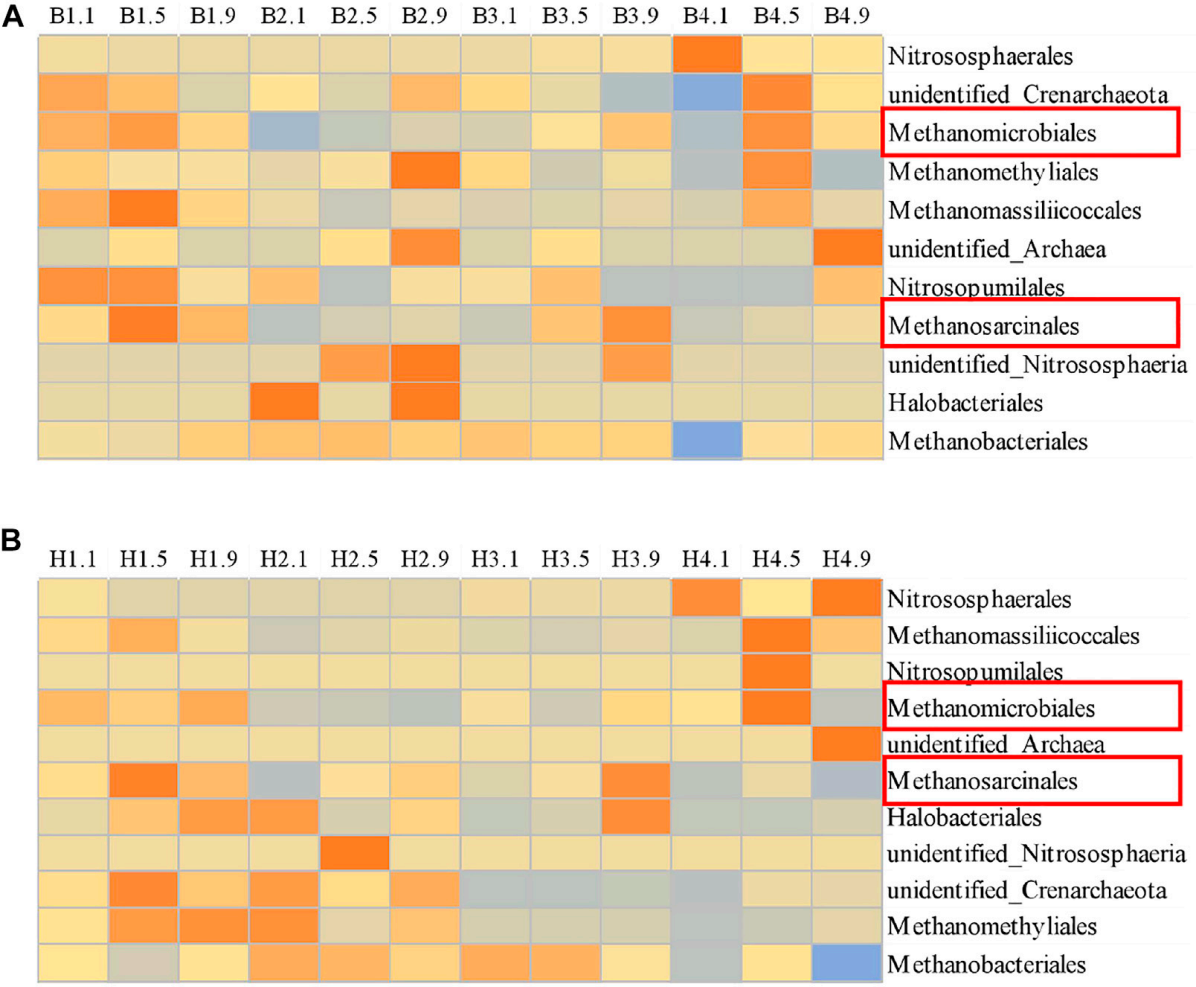
Composition of archaea community in each layer of CB **(A)** and CH **(B)**.

Aerobic methane oxidation in the landfill cover was considered to be the main way to achieve CH_4_ emission reduction (Chen et al., 2018; Wong et al., 2019), while anaerobic methane oxidation was ignored (Zhang et al., 2019). The low volume fraction of oxygen in the middle and lower layers of the landfill cover provided favorable conditions for the growth of anaerobic methanotrophic archaea (ANME) (Dong et al., 2015). The ANME with relatively high abundance detected in this study was *Methanosarcinales* and *Methanomicrobiales* (Figure 6). With the increase of CH_4_ concent in inlet, the relative abundance of *Methanosarcinales* in each sampling ports of CB and CH showed a decreasing trend (the color in the heat map gradually changed from red to blue (Figure 6), showing that it was not suitable for the environment of landfill cover–simulated columns. *Methanomicrobiales* was gradually decreased in the upper and lower layers of CB and CH, while it was enriched in the middle layers of CB and CH, indicating that ANME contributed to CH_4_ emission reduction in the middle layers of the landfill covers, but the addition of hydrophobic biochar had little impact on the abundance of ANME. In addition, some methanogenic archaea, such as *Methanobacteriales* and *Methanomassiliicoccales*, were found in both CH and CB, and mainly distributed in the middle and lower layers of CH and CB. The relative abundances of methanogenic archaea in the middle and lower layers of CH were 86.3 and 65.5%, respectively, both lower than 87.4 and 89.5% in CB. With these suggested that the middle and lower layers were the main methanogenic regions, where the methanogenic process of CH might be weaker than that of CB. It is evident that the cover layer simulation column is a dynamic CH_4_ production and consumption system. The reinforcement of ANME and suppression of methanogenic archaea are also essential for CH_4_ reduction.

### 3.4 Biological Mechanism of Hydrophobic Biochar–Amended Landfill Cover Soil

**FIGURE 7:**
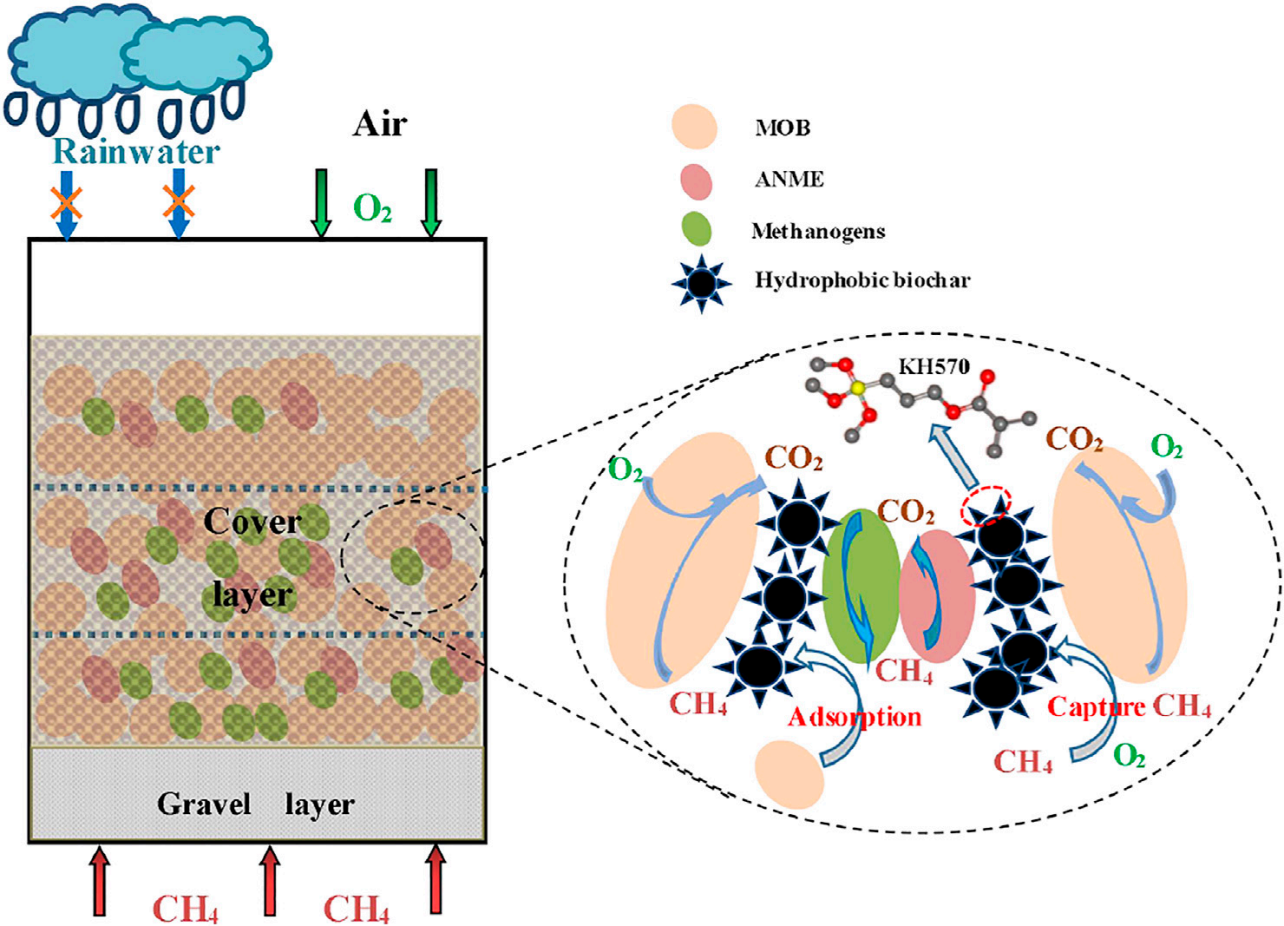
Biological mechanism diagram of HLCS.

The hydrophobic biochar replaced ordinary biochar was added to the landfill cover soil to form HLCS, the rainwater retention time in HLCS was reduced by half and an efficient CH_4_ reduction was achieved. Thus, HLCS can solve the contradiction between promoting the diffusion of CH_4_ and O_2_ and preventing rainwater from entering the cover layer, the biological mechanism as shown in Figure 7. The upper layer and lower layer of HLCS were the main parts responsible for CH_4_ emission reduction. The dominant MOB in HLCS was *Methylobacter*, and with high abundance in the lower and upper layers of HLCS. In addition, there was a certain abundance of ANME in the middle layer of HLCS, and some methanogenic archaea were also discovered in HLCS. It can be seen that the aerobic methane oxidation, anaerobic methane oxidation and methane production were all existed in HLCS. HLCS was a dynamic CH_4_ generation and consumption system, and the suppression of methanogenic archaea in HLCS is essential for CH_4_ emission reduction.

In addition, compared with BLCS, HLCS had an efficient CH_4_ reduction potential and higher abundance of MOB. These may be related to the higher zeta potential of hydrophobic biochar than ordinary biochar facilitated the adsorption of MOB (Afrooz et al., 2018; Ren et al., 2020). Meanwhile, the organic long chain (alkoxy group) of hydrophobic modifier KH570 grafted on biochar surface induced extensive agglomeration (Yang et al., 2012; Sun et al., 2019), which is conducive to the clustering of MOB and the capture of gas molecules such as CH_4_ and O_2_ (Zhang et al., 2019). More importantly, the large specific surface area and the appropriate moisture content of hydrophobic biochar provided good conditions for MOB growth (Yang et al., 2012; Reddy et al., 2014). Therefore, the cover layer of hydrophobic biochar soil had high abundance of MOB and thus showed relatively high CH_4_ oxidation efficiency.

## 4 Conclusion

Hydrophobic biochar amended to the landfill cover soil can realize waterproofing, ventilation, MOB growth promotion, and efficient CH_4_ reduction. The rainwater retention time in HLCS was reduced by half compared with BLCS. A completed removal of CH_4_ was achieved in HLCS with 25% CH_4_ content of the landfill gas. The main contributors to the CH_4_ removal in HLCS were found in depths 10–30 (upper layer) and 50–60 cm (lower layer), and the CH_4_ removal in its upper layer of HLCS was more effective than that in BLCS. The dominant MOB in HLCS was *Methylobacter*; the relative abundances of the MOB in the upper and lower layers of HLCS were significantly higher than those of BLCS. The ANME and methanogenic archaea were also discovered in HLCS, and the reinforcement of ANME and suppression of methanogenic archaea are also essential for CH_4_ reduction.

## Data Availability

The original contributions presented in the study are included in the article; further inquiries can be directed to the corresponding authors.
